# Excess heat production in the redox couple reaction of ferricyanide and ferrocyanide

**DOI:** 10.1038/s41598-020-76611-3

**Published:** 2020-11-18

**Authors:** Atsushi Sugiyama, Makoto Miura, Yoshinobu Oshikiri, Yena Kim, Ryoichi Morimoto, Miki Miura, Tetsuya Osaka, Iwao Mogi, Yusuke Yamauchi, Ryoichi Aogaki

**Affiliations:** 1 Yoshino Denka Kogyo, Inc, Yoshikawa, Saitama, 342-0008 Japan; 2 Research Organization for Nano and Life Innovation, Waseda University, Shinjuku, Tokyo, 162-0041 Japan; 3 JST-ERATO Yamauchi Materials Space-Tectonics Project and International Center for Materials Nanoarchitectonics (WPI-MANA), National Institute for Materials Science (NIMS), Tsukuba, Ibaraki, 305-0044 Japan; 4 Hokkaido Polytechnic College, Otaru, Hokkaido, 047-0292 Japan; 5 Yamagata College of Industry and Technology, Matsuei, Yamagata, 990-2473 Japan; 6 Saitama Industrial Technology Center, Kawaguchi, Saitama, 333-0844 Japan; 7 Polytechnic Center Kimitsu, Kimitsu, Chiba, 299-1142 Japan; 8 Institute for Materials Research, Tohoku University, Aoba-ku, Sendai, 980-8577 Japan; 9 School of Chemical Engineering and Australian Institute for Bioengineering and Nanotechnology (AIBN), The University of Queensland, Brisbane, QLD 4072 Australia; 10 Polytechnic University, Sumida, Tokyo, 130-0026 Japan

**Keywords:** Chemistry, Engineering

## Abstract

In order to establish the universality of the excess heat production in electrochemical reaction, under a high magnetic field, as one of the most fundamental electrochemical reactions, the case of ferricyanide-ferrocyanide redox reaction was examined, where ionic vacancies with ± 1 unit charge were collided by means of magnetohydrodynamic (MHD) flow. As a result, from the pair annihilation of the vacancies with opposite signs, beyond 7 T, excess heat production up to 25 kJ·mol^−1^ in average at 15 T was observed, which was attributed to the liberation of the solvation energy stored in a pair of the vacancy cores with a 0.32 nm radius, i.e., 112 kJ·mol^−1^. Difference between the observed and expected energies comes from the small collision efficiency of 0.22 due to small radius of the vacancy core. Ionic vacancy initially created as a by-product of electrode reaction is unstable in solution phase, stabilized by releasing solvation energy. Ionic vacancy utilizes the energy to enlarge the core and stores the energy in it. As a result, solvated ionic vacancy consists of a polarized free space of the enlarged core surrounded by oppositely charged ionic cloud. The accuracy and precision of the measured values were ascertained by in situ standard additive method.

## Introduction

**Figure 1:**
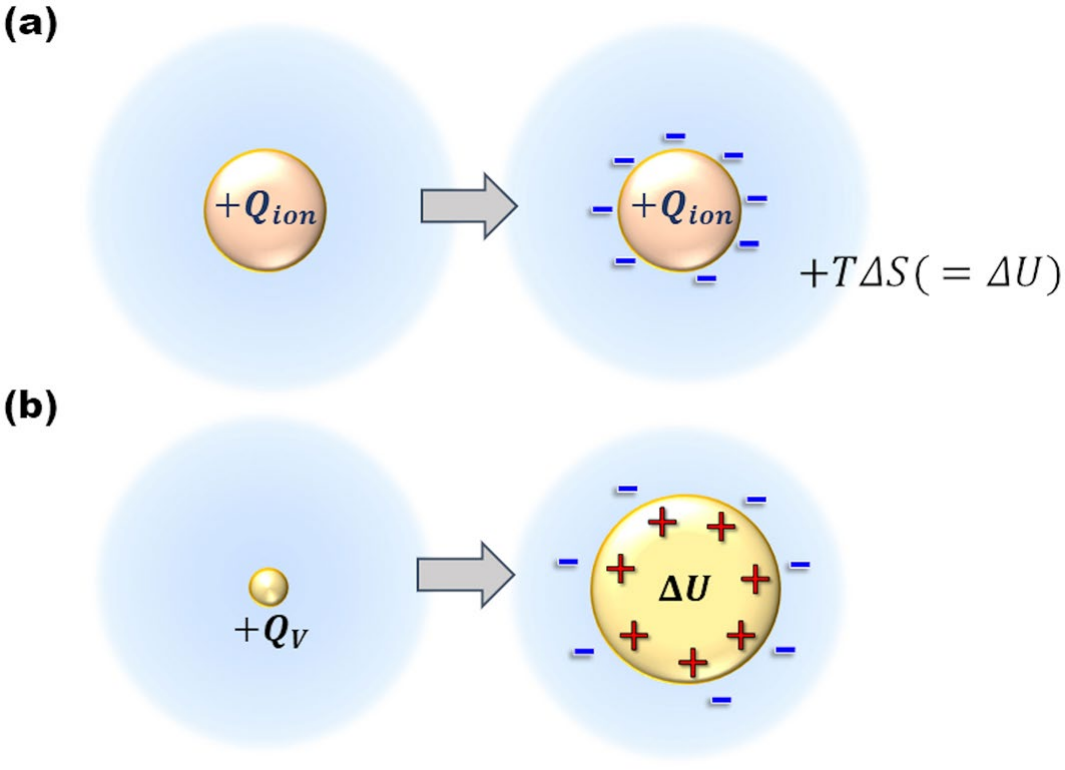
Solvations of isolated ion and embryo vacancy. (**a**) In case of ion (cation): Entropy is produced by the solvation energy emitted from the ionic cloud. $+ Q_{ion}$, ionic charge of cation; ${\Delta }S$, produced entropy; $T$, absolute temperature. (**b**) In case of positive ionic vacancy: Instead of entropy production, the vacancy core is expanded by the solvation energy, i.e., it is stored in the vacancy core. $+ Q_{V}$, polarized charge of positive vacancy; ${\Delta }U$, solvation energy stored in vacancy core.

Recently, in copper redox reaction under a high magnetic field, great excess heat production up to 410 kJ·mol^−1^ in average has been observed, which is 1.5 times larger than the molar combustion heat of hydrogen (285.8 kJ·mol^−1^)^1^. It was attributed to the pair annihilation of ionic vacancies with $\pm 2$ unit charges created in copper cathodic and anodic reactions. Ionic vacancy is created with the electron transfer in electrode reaction. To conserve the linear momentum and electric charges, polarized embryo vacancy is emitted to solution phase^2^. Since a charged particle like isolated ion is energetically unstable in solution, it is quickly solvated, surrounded by ionic cloud with solvation energy released. As shown in Fig. 1a,b, using the solvation energy, ion produces entropy, whereas embryo vacancy enlarges its core, storing the energy in it.

**Figure 2:**
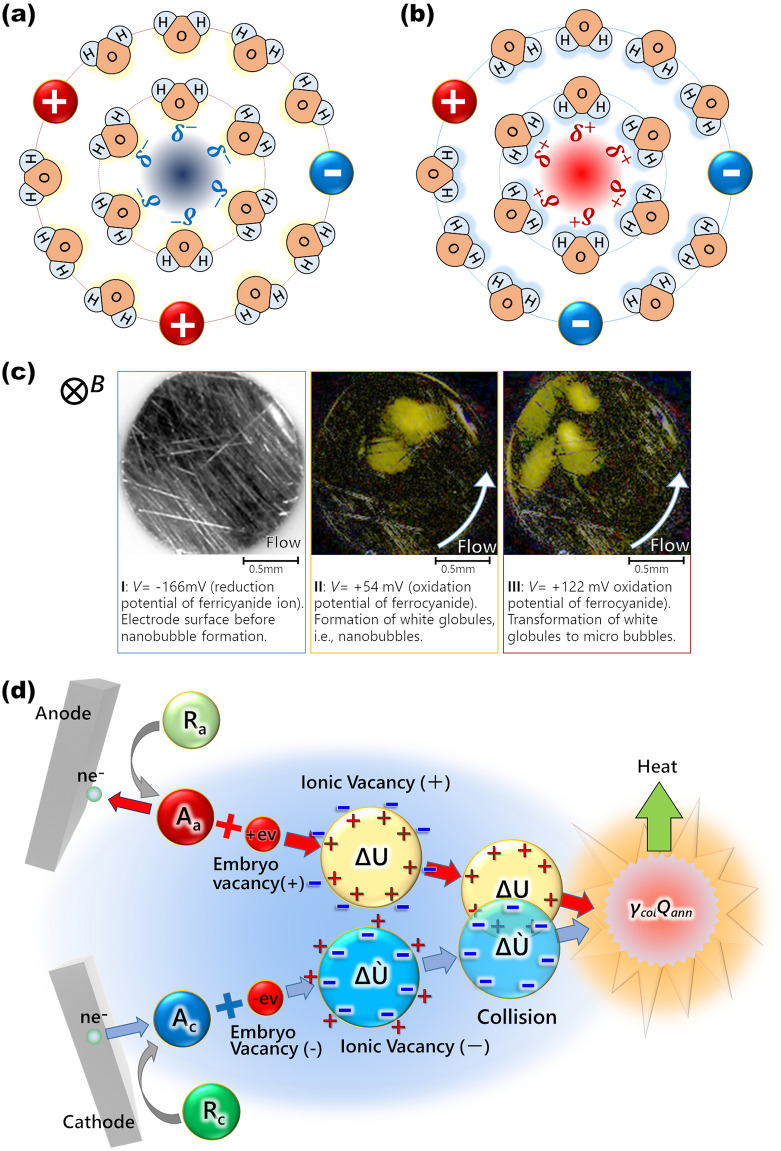
Pair annihilation of ionic vacancies with opposite charges. (**a**) Schematic of a vacancy core with negative polarized charge formed in water solution. H, hydrogen atom; O, oxygen atom; $\delta^{ - }$, partial polarized charge of the water molecule surrounding the free space; $\ominus$, anion; $\oplus$, cation. (**b)** Schematic of a vacancy core with positive polarized charge formed in water solution. H, hydrogen atom; O, oxygen atom; $\delta^{ + }$, partial polarized charge of the water molecule surrounding the free space; $\ominus$, anion; $\oplus$, cation. (**c**) Microbubble evolution on the electrode in ferrocyanide oxidation under a 8 T magnetic field (modified^5^). *V*, overpotentials from the rest potential (*V* =  − 166 mV, + 54 mV, + 122 mV corresponding to the electrode potentials, E =  + 264 mV, + 484 mV, + 552 mV vs. NHE, respectively); [K_4_(Fe(CN)_6_)], 300 mol·m^−3^; [K_3_(Fe(CN)_6_)], 100 mol·m^−3^; [KCl], 500 mol·m^−3^. (**d**) Pair annihilation of positive and negative vacancies. *n*e^−^, transferring electric charges of electrons; R_a_, anodic reactant; R_c_, cathodic reactant; A_a_, anodic activated complex; A_c_, cathodic activated complex; + ev, positive embryo vacancy; −ev, negative embryo vacancy; ${\Delta }U$, stored solvation energy; $\gamma_{col} Q_{ann}$, molar excess heat.

Solvated ionic vacancy is, as shown in Fig. 2a,b, composed of a positively or negatively polarized free space of the order of 0.1 nm (vacancy core) surrounded by oppositely charged ionic cloud^3^. Due to the smallness and a lifetime of 1 s^4^, it is difficult to observe the solvated vacancies directly but easy by magnetic field. In the electrolysis under a vertical magnetic field, a tornado-like rotation called vertical magnetohydrodynamic (MHD) flow emerges over a disk electrode [vertical MHD electrode (VMHDE)]. Under the rotation, a radial secondary flow is induced, so that created ionic vacancies with the same sign are conveyed to the electrode center, forming a vacancy layer, where they are collided with each other, yielding nanobubbles. Furthermore, the nanobubbles are changed to microbubbles by further collisions. Figure 2c exhibits the microbubble clusters observed in ferricyanide-ferrocyanide redox reaction formed on a platinum VMHDE^5^. Such a cluster of microbubbles have been also observed in copper cathodic deposition^6^ and copper anodic dissolution^7^.

Chemical nature of ionic vacancy was examined by the adsorption onto copper three-dimensional (3D) nuclei, blocking the nucleation, which, unrelated to hydrogen gas evolution, gives rise to characteristic copper dendrite (Magneto-dendrite effect)^8^. Then, the chemical reactions of ionic vacancies, i.e., collisions of ionic vacancies were examined: As mentioned above, the collision between ionic vacancies with the same sign yields nanobubbles^9^. On the other hand, in the case of collision between ionic vacancies with opposite signs, after neutralization of surrounding ionic clouds, the depolarized vacancy cores are annihilated, releasing the stored solvation energies as excess heat (Fig. 2d). The excess heat production in copper redox reaction was measured by a MHD electrode system [circulation-type MHD electrode (c-type MHDE)]^1^, referring to the calorimetry experiment in lithium batteries^10,11^.

As well known in magnetoelectrochemistry, MHD electrode provides useful methods for the reaction analysis in magnetic field^12–21^. The magnetically assisted electrolysis is operated under a magnetic field parallel to electrode surface. Lorentz force induces a solution flow called MHD flow promoting mass transport of ions. For the MHD flow in a parallel magnetic field, MHD-pumping electrode cell called MHD electrode (MHDE) was developed^22–24^, where the velocity and concentration distribution reduce to the simple equations of the velocity and diffusion current, which provides excellent agreement between theory and experimental result^16^. A notable advantage of this type of electrode lies in the practical possibility of using very small cells without mechanical means. In high magnetic field, the strong stirring by Lorentz force easily attains isothermal condition.

In copper redox reaction in the foregoing paper^1^, vacancies with $\pm$2 unit charges are created, and the excess heat production attained up to 410 kJ·mol^−1^ in average. Such a large amount of excess heat can be measured by the curve-fitting method based on Ohm’s law, where we firstly applied sweeping current to a c-type MHDE and measured the rise of solution temperature. Then, by means of Ohm’s law, the temperature was expressed by the 3rd order of equation of the current. Finally, from fitting the equation to the regression curve of the experimental data, the excess heat was determined.

However, in the case of a redox reaction creating vacancies with $\pm$ 1 unit charges such as ferricyanide-ferrocyanide redox reaction, the excess heat is estimated much smaller than that of the case of $\pm$ 2 unit charges. To measure such a small amount of excess heat, more precise method is required, i.e., in the measurement of excess heat, whether the isothermal condition is fulfilled or not is quite important. Moreover, if possible, to estimate the accuracy and precision of the measurement, the calibration of the measurement with a reference material is also necessary.

To experimentally prove the universality of the excess heat production by the pair annihilation of ionic vacancies, following copper redox reaction, in the present paper, focusing on one of the most fundamental electrode reactions, ferricyanide–ferrocyanide redox reaction, we measure the excess heat by adopting a new method called Joule’s heat capacity method.

## Results

### Theory

The equilibrium potential of ferricyanide–ferrocyanide redox reaction is more anodic than the hydrogen evolution potential as well as more cathodic than the oxygen evolution potential. Therefore, in the absence of hydrogen and oxygen evolution, the ferricyanide cathodic reaction and ferrocyanide anodic reaction involving vacancy production are simply expressed as follows,

1 $$\left[ {Fe\left( {CN} \right)_{6} } \right]^{ 3-} + e^{ - } \to \left[ {Fe\left( {CN} \right)_{6} } \right]^{ 4-} + V_{ - } \left( {cathodic\,reaction} \right),$$

2 $$\left[ {Fe\left( {CN} \right)_{6} } \right]^{ 4-} - e^{ - } \to \left[ {Fe\left( {CN} \right)_{6} } \right]^{ 3-} + V_{ + } \left( {anodic\,reaction} \right),$$

where $V_{ - }$ and $V_{ + }$ depict the vacancies with $-$ 1 and $+$ 1 unit charges, respectively. In the electrode reaction, an electron transfers between the electrode and the reactant. At the same time, the momentum and charge of the electron also transfer to the solution phase, yielding a polarized vacancy^2^. As a result, in case of the transfer from the electrode to the reactant (reduction), negative vacancy $V_{ - }$ is created, whereas for the transfer from the reactant to the electrode (oxidation), positive vacancy $V_{ + }$ emerges. In the isolated state of stationary solution, whether the charges are the same or not, individual ionic vacancies reversibly disappear without any heat production. However, in case of the collision of the vacancies with opposite charges, as shown in Fig. 2d_,_ the neutralization of ionic clouds would lead to the following pair annihilation,

3 $$V_{ - } + V_{ + } \to Null + \gamma_{col} Q_{ann} ,$$

where $\gamma_{col}$ is the collision efficiency, and $Q_{ann}$ is the molar excess heat (J·mol^−1^) liberated from the stored solvation energy by the annihilation. The molar heat production of the system by the pair annihilation is therefore given by^1^

4 $$Q_{ann} = 8\pi N_{A} \sigma R^{*2} ,$$

where $N_{A}$ is the Avogadro number, $R^{*}$ is the core radius (m), and $\sigma$ is the surface tension of water (J·m^−2^). Since the theoretical core radii of the vacancies with one and two unit charges are calculated as $R^{*}$ = 0.32 nm and 0.65 nm , respectively^3^, with $N_{A}$ = 6 $\times$ 10^23^ mol^−1^ and $\sigma$ = 7.2 $\times$ 10^–2^ J·m^−2^ (surface tension of water at 25 °C), the molar energies stored in the vacancies with one and two unit charges are estimated as 56 and 229 kJ·mol^−1^, respectively. We can therefore expect the molar excess heat production by the pair annihilation of the vacancies with $\pm$ 1 and $\pm$ 2 unit charges as 112 and 456 kJ·mol^−1^, respectively. Due to the smallness of the vacancy with $\pm$ 1 unit charge, the collision efficiency $\gamma_{col}$ may be not so high that the actual heat emission from the ferricyanide-ferrocyanide reaction is expected to some extent lower than the theoretical value.

**Figure 3:**
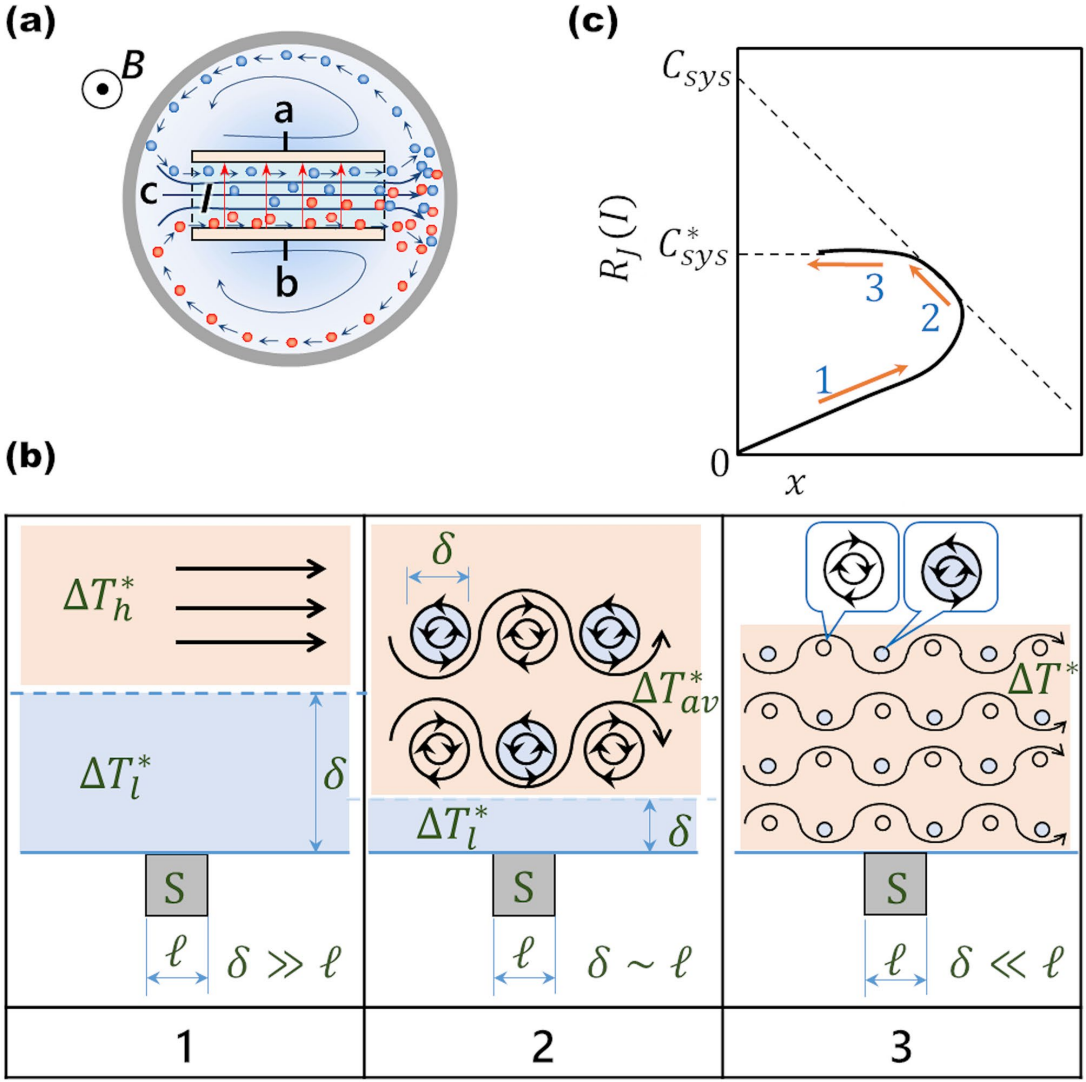
Measurement of excess heat production by c-type MHDE. (**a**) MHD flow and ionic vacancies created by c-type MHDE. a, Cathode; b, Anode; c, Streamlines; *I*, Electrolytic current; *B*, Magnetic flux density; blue circle, Negative vacancy; red circle, Positive vacancy. (**b**) MHD flow pattern around a thermal sensor. (1) Low current range (two-temperature-phase state); (2) Middle current range (quasi-isothermal state); (3) High current range (isothermal state). S, thermal sensor; $\ell$, size of the thermal sensor; $\delta$, thickness of the low-temperature phase. $\Delta T_{l}^{*}$, temperature difference of the low-temperature phase; $\Delta T_{h}^{*}$, temperature difference of the high-temperature phase; $\Delta T_{av}^{*}$, average temperature difference of the mixed solution; ${\Delta }T^{*}$, temperature difference of the unified solution under a isothermal condition. (**c**) Schematic of the locus of $R_{J} \left( I \right)$ vs. $x$. (1) Low current range; (2) Middle current range; (3) High current range. $C_{sys}$, calorimeter constant in the middle current range; $C_{sys}^{*}$, calorimeter constant in the high current range.

Figure 3a shows a schematic of c-type MHDE. A pair of platinum electrodes are imbedded face to face as cathode and anode on the inner walls of a rectangular channel with two open ends. The whole electrode is settled in a narrow vessel. Under a parallel magnetic field, electrolytic current induces MHD flow by Lorentz force, carrying out of the channel the vacancies created on the electrodes to collide at the vessel wall. Due to narrow space of the vessel, the vacancies escaping from the collision quickly participate the collision again, which enhances the collision efficiency.

### Joule’s heat capacity method for electrochemical calorimetry experiment

To calculate the reaction heat by the pair annihilation, we apply a positive current *I* (A) increasing with time* t* (s) to a c-type MHDE.

5 $$I = at{\text{ for }}t \ge 0,$$

where $a$ is the positive sweep rate (A·s^−1^). As discussed in Supplementary Appendix A, the equation of heat balance in the electrode system is expressed by

6 $$C_{sys} \Delta T^{*} \left( { = Q_{tot} } \right) = \frac{1}{a}\mathop \smallint \limits_{0}^{I} \Delta VIdI + \frac{{\gamma_{col} Q_{ann} }}{2nFa}I^{2}\, {\text{for}} \,I \ge 0,$$

where $C_{sys}$ is the calorimeter constant, i.e., the effective heat capacity of the system (J·K^−1^), $\Delta T^{*}$ is the difference between the solution and ambient temperatures compensated for escaping heat (K) used as $\Delta T_l^{*}$ Supplementaly Eq. (A.11) in Supplementaly Appendix A, and $Q_{tot}$ is the total heat stored in the cell system (J·mol^−1^). $\Delta V$ (> 0) is the cell voltage (V), $\gamma_{col}$ is the collision efficiency, and $Q_{ann}$ is the molar excess heat (J·mol^−1^). Then, $n$ is the electron number transferring in the cell reaction, and $F$ is Faraday constant (96,485 C·mol^−1^).

Measurement of the quantity of heat shown in Eq. (6) must be carried out under isothermal condition. However, for the in-situ measurement of reaction heat, since the measuring point of thermal sensor is apart from the heat source in front of the channel, being set on the outer wall of the channel, it is difficult to always achieve isothermal condition. In the vessel containing a MHDE, the solution is stirred by the MHD flow, which seriously affects the value measured by the thermal sensor. To establish the criteria of suitable measurement, as shown in Fig. 3b, we introduce a simple model of a measurement system including heat source, thermal sensor, solution and vessel.

1. The solution is initially composed of isothermal high- and low-temperature phases.
2. The heat source belongs to the high-temperature phase and the thermal sensor is involved in the low-tenperature phase.
3. The thickness of the low-temperature phase $\delta$ and the size of the sensor $\ell$ are introduced.

In accordance with this model, sweeping the electrolytic current, we can observe the following three ranges of the current: In the range of low current, the MHD flow is laminar and so weak that macroscopic separation of the two phases with different temperatures is maintained (two-temperature-phase state, Fig. 3b(1)), where the relation of the order of length $\delta \gg \ell$ is fulfilled. However, in the range of middle current, the MHD flow changes to a transient flow with Kármán’s vortexes^25^, and the mixing of the two phases proceeds (quasi-isothermal state, Fig. 3b(2)), where the two phases still coexist in numerous small parts of the order of length of $\ell$, and the thickness of the low temperature phase $\delta$ also decreases to the order of $\ell$, i.e., $\delta \approx \ell$. As the current furthermore increases, in the range of high current, due to turbulent flow, perfect molecular-level mixing finally occurs (isothermal state, Fig. 3b(3)), i.e., $\delta \ll \ell$.

As a measurable quantity, we define the following Joule’s heat capacity $R_{J} \left( I \right)$ (J·K^−1^).

7 $$R_{J} \left( I \right) \equiv Q_{Joule} /\Delta T_{l}^{*}\, {\text{for}} \,\Delta T_{l}^{*} > 0,$$

where $\Delta T_{l}^{*}$ (K) is the temperature difference measured by thermal sensor in the low temperature phase. Here, it should be noted that $\Delta T_{l}^{*}$ is only a measurable temperature by the thermal sensor. $Q_{Joule}$ (J) is Joule’s heat defined by

8 $$Q_{Joule} \equiv \frac{1}{a}\mathop \smallint \limits_{0}^{I} \Delta VIdI$$

and the excess heat production $Q_{excess}$ (J) is expressed by

9 $$Q_{excess} \equiv \frac{{\gamma_{col} Q_{ann} }}{2nFa}I^{2} .$$

Then, considering that the excess heat in Eq. (9) is in proportion to the 2nd power of the current $I^{2}$, as another measurable quantity, we introduce the variable $x$ (A^2·^K^−1^).

10 $$x\; \equiv \;I^{2} /\Delta T_{l}^{*} .$$

Using $R_{J} \left( I \right)$ and $x$, we make the plot of $R_{J} \left( I \right)$ against $x$, of which locus is classified in three current ranges.

#### Low current range (two-temperature-phase state)

In the early stage of current sweep, small electrolytic current induces a weak laminar MHD flow (Fig. 3b(1)), leading to small collision efficiency of the vacancies, i.e., $\gamma_{col} \approx 0$, and the resultant small excess heat production, $Q_{excess} \approx 0$. The heat production of the heat source in the high-temperature phase thus comes from Joule’s heat, and due to weak laminar flow, the low-temperature phase including thermal sensor is still maintained. Namely, in the low current range, we cannot measure the excess heat $Q_{excess}$. As a result, as shown in Supplementary Appendix B, $R_{J} \left( I \right)$ is approximately expressed by the linear equation of *x* with a positive slope.

11 $$R_{J} \left( I \right) \approx C_{sys,l} + Q_{h}^{*} x,$$

where $C_{sys,l}$ is the calorimeter constant of the low-temperature phase, and the slope $Q_{h}^{*}$ (J·A^−2^) is approximately proportional to $I$,

12 $$Q_{h}^{*} \approx Q_{Joule} /I^{2} \propto I ( > 0).$$

As discussed in Supplementary Eq. (B.23) in Supplementary Appendix B, since $Q_{Joule}$ depends on the 3rd power of $I$, $Q_{h}^{*}$ increases with $I$. On the other hand, due to weak MHD flow, the low-temperature phase hardly receives the heat, so that $\Delta T_{l}^{*}$ is approximately kept constant. From Eq. (10), this means that the variable* x* increases with $I2$. Namely, in the low current range, it is expected that the locus of $R_{J} \left( I \right)$ against $x$ draws a rising curve.

#### Middle current range (quasi-isothermal state)

With current increasing, Lorentz force is strengthened, so that the laminar solution flow is changed into a transient flow with Kármán’s vortexes shown in Fig. 3b(2). By the mixing of the solution, the collision efficiency of vacancies $\gamma_{col}$ increases, so that the excess heat production $Q_{excess}$ in Eq. (9) by the pair annihilation is greatly promoted. The mixture however does not attain the perfect molecular mixing, but is kept in a quasi-molecular mixing state composed of numerous low- and high-temperature sub-phases with small volumes of the scale of length $\ell$*.* Since the heat transfer between the sub-phases is much faster than the rise of measured temperature, the whole system is approximately regarded isothermal. In this quasi-isothermal state, owing to the scale of length of the sub-phases approximately equal to the size of sensor $\ell$, we can treat the cell system in the same way as the system in perfect isothermal state, so that we obtain the following equation.

13 $$R_{J} \left( I \right) = C_{sys} - \frac{{\gamma_{col} Q_{anni} }}{2nFa}x,$$

where $C_{sys}$ is the calorimeter constant of the system in the quasi-isothermal state.

As the MHD flow is promoted, the collisions of ionic vacancies with opposite charges drastically increase. The excess heat production is activated together with the mixing between the two phases, so that the temperature difference $\Delta T_{l}^{\;*}$ of the low-temperature phase involving thermal sensor greatly increases, and the variable $x$ ($\equiv I^{2} /\Delta T_{l}^{\;*}$) decreases. Due to the linear function of $x$ with a negative slope in Eq. (13), $R_{J} \left( I \right)$ increases with decreasing $x$. Namely, in the middle current range, the locus of $R_{J} \left( I \right)$ draws a straight line with a negative slope of $- \gamma_{col} Q_{ann} /\left( {2nFa} \right)$, of which the intercept at the $R_{J} \left( I \right)$-axis is equal to the calorimeter constant $C_{sys}$ in the quasi-isothermal state.

#### High current range (isothermal state)

In the high current range, as the Lorentz force increases, as shown in Fig. 3b(3), the transient MHD flow is furthermore changed into turbulent flow with numerous micro-vortexes. As a result, a perfect mixing state, i.e., a perfect isothermal state emerges, where the measured temperature difference $\Delta T_{l}^{\;*}$ in the low-temperature phase and also the temperature difference $\Delta T_{h}^{\;*}$ in the high-temperature phase become equal to that of the isothermal phase $\Delta T^{*}$. As discussed in Supplementary Eq. (B.23) in Supplementary Appendix B, in the high current range, Joule’s heat becomes dominant again, so that with decreasing $x$*,* the locus of $R_{J} \left( I \right)$ approaches a constant value of the calorimeter constant of the system in the isothermal state, $C_{sys}^{*}$.

14 $$R_{J} \left( I \right) = C_{sys}^{*} .$$

That is, in the high current range, we cannot measure the excess heat production again. In Fig. 3c, the whole locus of $R_{J} \left( I \right)$ discussed here is schematically exhibited.

### Examination of the accuracy and precision of this method

As discussed in Supplementary Appendix C, the effective specific heat $c_{eff,hc}$ measured in the high current range is consistent with the specific heat of the experimental solution $c_{sol}$. As a result, in terms of the standard additive method by adopting the solution used in the experiment containing electrolyte, i.e., a 300 mol·m^−3^ K_3_[Fe(CN)_6_] + 300 mol·m^−3^ K_4_[Fe(CN)_6_] + 100 mol·m^−3^ KCl solution as a reference material, we can in situ examine the accuracy and precision of the measurement. Here, the effect of the addition of electrolyte on the specific heat of water is so small that the specific heat of water containing a large amount of electrolyte, i.e., a 700 mol·m^−3^ KCl solution is 3.916 J g^−1^ K^−1^ at 25 °C, whereas that of pure water is 4.179 J·g^−1^·K^−1^ at 25 °C, of which difference is only 6%. Therefore, instead of the actual heat capacity of the solution, we can compare the obtained heat capacity with these two data as standard values.

**Figure 4:**
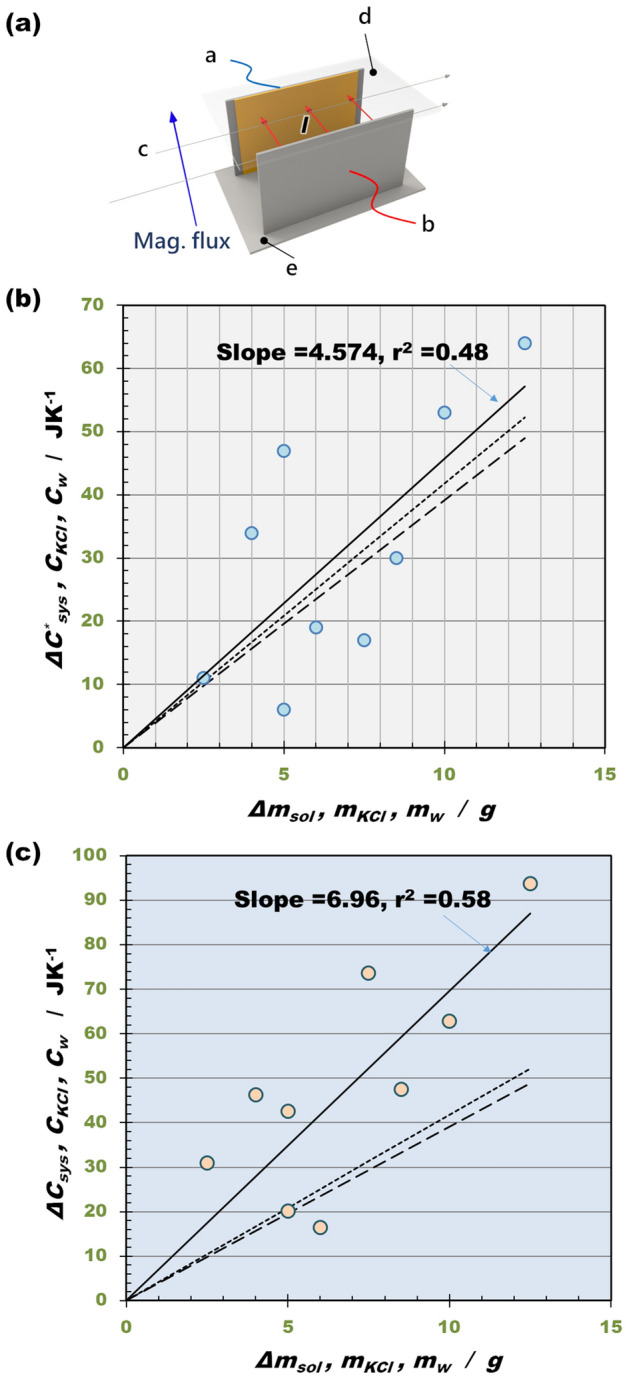
Comparison of the effective heat capacities ${\Delta }C_{sys}$ and ${\Delta }C_{sys}^{*}$ against the added solution mass. (**a**) The apparatus of a c-type MHDE. a, cathode; b, anode; c, stream line of electrolyte; d, upper wall; e, bottom wall. (**b**) Plot of the measured effective heat capacity in the high current range vs. the added solution mass. ${\Delta }C_{sys}^{*}$ is the effective heat capacity (= $c_{eff,hc} \Delta m_{sol}$) of a 300 mol·m^−3^ K_3_[Fe(CN)_6_] + 300 mol·m^−3^ K_4_[Fe(CN)_6_] + 100 mol·m^−3^ KCl solution in the high current range, where $c_{eff,hc}$ is the effective specific heat of the solution in the high current range, and ${\Delta }m_{sol}$ is the added solution mass. Solid line is the linear regression of the data of the effective heat capacity. Break line is the plot of the heat capacity of a 700 mol·m^−3^ KCl solution $C_{KCl}$ at 25 °C (= $c_{KCl} m_{KCl}$), where $c_{KCl}$ is the specific heat of a 700 mol·m^−3^ KCl solution (= 3.916 J·g^−1^ K^−1^ at 25 °C), and $m_{KCl}$ is the mass of the KCl solution. Dotted line is the plot of the heat capacity of pure water $C_{w}$ at 25 °C (= $c_{w} m_{w}$), where $c_{w}$ is the specific heat of pure water (= 4.179 J g^−1^ K^−1^ at 25 °C), and $m_{w}$ is the water mass. (**c**) Plot of the effective heat capacities of the solution in the middle current range vs. the added solution mass. ${\Delta }C_{sys}$ is the effective heat capacity (= $c_{eff,mc} \Delta m_{sol}$) of a 300 mol·m^−3^ K_3_[Fe(CN)_6_] + 300 mol·m^−3^ K_4_[Fe(CN)_6_] + 100 mol·m^−3^ KCl solution in the middle current range, where $c_{eff,mc}$ is the effective specific heat of the solution in the middle current range. Solid line is the linear regression of the data of the measured effective heat capacity. Break line is the heat capacities of a 700 mol·m^−3^ KCl solution $C_{KCl}$ at 25 °C. Dotted line is the plot of the heat capacity of pure water $C_{w}$ at 25 °C. The sweep rate of the current is a = 0.05 mA·s^−1^.

Figure 4a exhibits the experimental apparatus of c-type MHDE, which was settled on the bottom of a long glass tube with a 6.5 cm length and a 3.7 cm diameter. The whole tube was inserted in the bore of 15 T superconducting magnet. To prevent natural convection, the bore temperature was, as much as possible, controlled to quite slowly decrease with time (see Supplementary Appendix A). After drawing the locus of Joule’s heat capacity $R_{J} \left( I \right)$, the calorimeter constants $C_{sys}$ and $C_{sys}^{*}$ in the middle and high current ranges were calculated.

In accordance with Supplementary Appendix C, measuring the increment of the calorimeter constant $\Delta C_{sys}^{*}$ against newly added mass $\Delta m_{sol}$ (g) of the solution from the Joule’s heat capacity plot in the high current range, we can determine the heat capacity of the added solution. As shown in Fig. 4b, the heat capacity $\Delta C_{sys}^{*}$ is in good agreement with the standard values of the heat capacities of a 700 mol·m^−3^ KCl solution $C_{KCl}$ and pure water $C_{w}$. From the experimental data shown in Fig. 4b, by means of Supplementary Eqs. (C.1) and (C.2), we derived the accuracy i.e., the degree of the agreement of the average value with the standard value, $\varepsilon_{a}$ = 78.3% and the precision, i.e., the degree of the deviation from the average value $\sigma_{p}$ = 65.4% for a 700 mol·m^−3^ KCl solution, whereas for pure water, $\varepsilon_{a}$ = 86.0% and $\sigma_{p}$ = 61.3% are derived. Namely, though the deviation from the average value is large (61.3 $\sim$ 65.4%), the agreement of the average value with the standard values is high (78.3 $\sim$ 86.0%). This implies that the measurement has a sufficient accuracy to measure the excess heat by the pair annihilation.

Then, from the increment of the calorimeter constant $\Delta C_{sys}$ against the added mass of the experimental solution in the middle current range, we can extract the effective specific heat $c_{eff,mc} \left( { = \Delta C_{sys} /\Delta m_{sol} } \right)$ in the middle current range, of which ratio against the standard value of the specific heat is, as shown in Supplementary Appendix C, equal to the ratio of the average temperature difference $\Delta T_{av}^{*}$ against the temperature difference of the low-temperature phase $\Delta T_{l}^{*}$ in the middle current range (see Supplementary Eq. (C.23)). Figure 4c represents the plot of the measured heat capacity $\Delta C_{sys}$ vs. the added mass $\Delta m_{sol}$ (g) of the experimental solution, of which slope is equal to the effective specific heat $c_{eff,mc}$ in the middle current range. In the same way, the slopes of the heat capacities $C_{KCl}$ and $C_{w}$ represent the specific heats $c_{KCl}$ and $c_{w}$ as the standard values. From the experimental data, it is concluded that the temperature difference ratio $\Delta T_{av}^{*} /\Delta T_{l}^{*}$ = 1.22 for a 700 mol·m^−3^ KCl solution, and $\Delta T_{av}^{*} /\Delta T_{l}^{*}$ = 1.14 for pure water, i.e., the average temperature difference $\Delta T_{av}^{*}$ in the middle current range is 1.14 $\sim$ 1.22 times higher than the temperature difference of the low-temperature phase $\Delta T_{l}^{*}$ which is actually measured in the middle current range. As discussed in Supplementary Appendix C, this is the experimental evidence of the existence of the quasi-isothermal state in the middle current range.

### Excess heat evolution in [Fe(CN)_6_]^3^^−^/[Fe(CN)_6_]^4^^−^ electrochemical system

**Figure 5:**
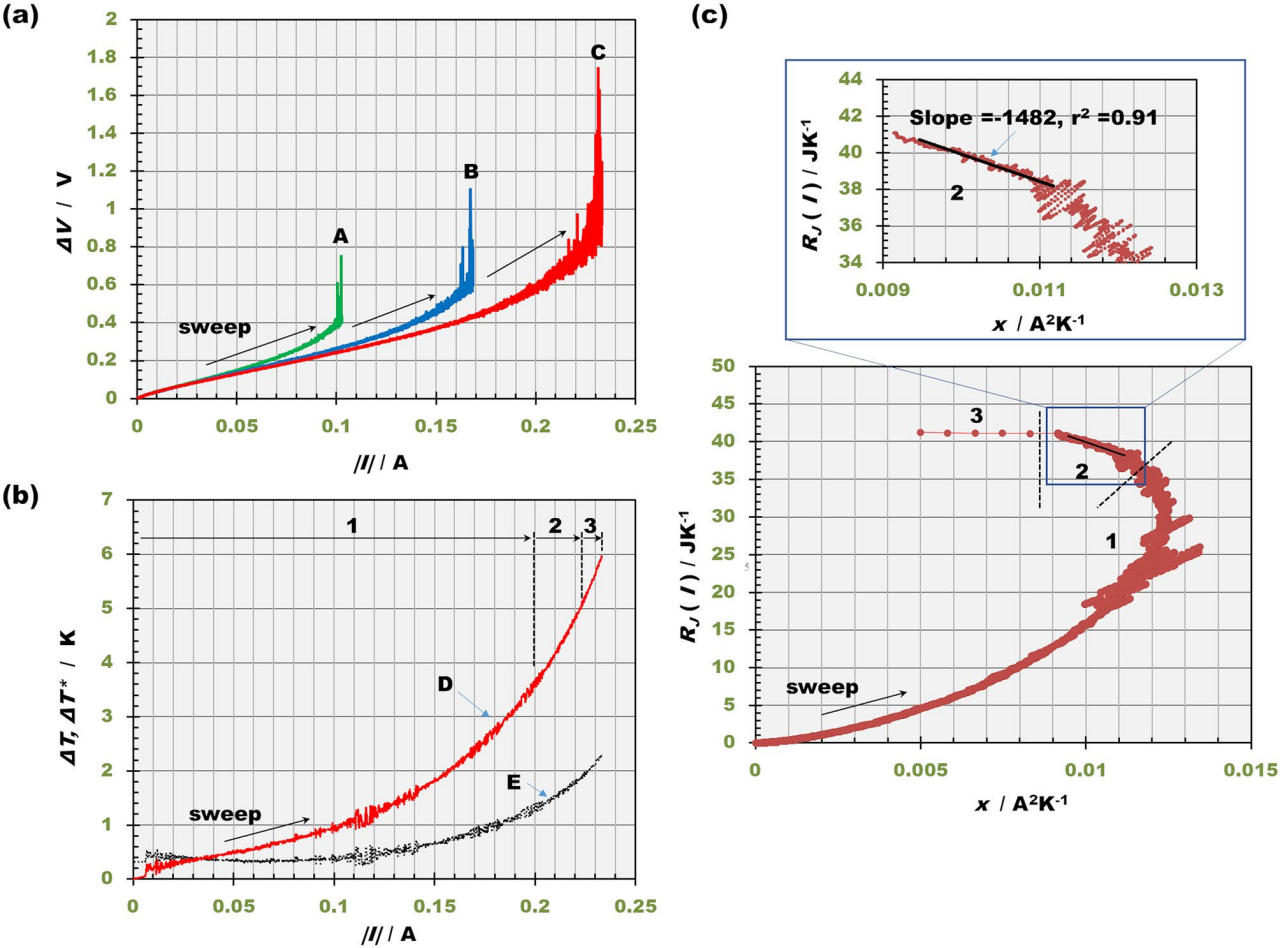
Temperature responses against the current sweeps under high magnetic fields. (**a**) Cell voltage response against current sweep in a rate of 0.05 mA·s^−1^. Green line A, at 5 T; blue line B, at 10 T; red line C, at 15 T. (**b**) Compensated temperature difference $\Delta T^{*}$ vs. current $I$ plot at 15 T. For comparison, the direct temperature difference $\Delta T$ measured during the experiment is also plotted. Red line D is the compensated temperature difference, and black line E is the direct temperature difference. The numbers 1, 2 and 3 correspond to those in the locus of $R_{J} \left( I \right)$. (**c**) Locus of $R_{J} \left( I \right)$ vs. $x$ in ferricyanide-ferrocyanide redox reaction at 15 T. (1) Low current range; (2) middle current range; (3) high current range. The linear regression of the data in the middle current range is shown in the inset. The volume of the solution is *v* = 7.5 mL, and the sweep rate of the current is a = 0.05 mA·s^−1^.[K_3_[Fe(CN)_6_]] = 300·mol·m^−3^; [K_4_[Fe(CN)_6_]] = 300 mol·m^−3^; [KCl] = 100 mol·m^−3^.

Figure 5a represents the response of the cell voltage $\Delta V$ against the sweeping current $I$ in a rate of 0.05 mA·s^−1^ at 5 T, 10 T and 15 T. At each upper limit of current, the cell voltage $\Delta V$ sharply rises until the decomposition potential of water. Due to the enhancement of mass transfer by MHD flow, the upper limit increases with the magnetic flux density.

Due to the heat escaping from the cell system, the temperature difference decreases. To compensate it, after switching off the current at the upper limit, we recorded the decreasing temperature difference. Then, according to the foregoing paper^1^, we plotted the recorded temperature difference against time in semi-log scale. Finally, from the slopes of the plot, the time constant $\alpha$ of the escaping heat was calculated. The average value of the time constant was $\alpha$ = 7.24 $\times$ 10^–4^ $\pm$ 9.8 $\times$ 10^–5^ s^−1^. From the measured temperature difference $\Delta T$ and each value of $\alpha$, using Supplementary Eq. (A.11) in Supplementary Appendix A, we determined the compensated temperature difference $\Delta T^{*}$ with the increasing current $I$ at a given magnetic flux density $B$. Figure 5b exhibits the plot of the compensated total temperature difference $\Delta T^{*}$ vs. current $I$ at 15 T. For comparison, the temperature difference $\Delta T$ directly measured by thermal sensor is also plotted. At the upper limit of 0.2 A, the compensated temperature difference attains $\Delta T^{*} \approx$ 6.0 K, whereas the direct temperature difference is $\Delta T \approx$ 2.3 K. The numbers 1, 2 and 3 in the plot correspond to the low current range, middle current range and high current range, respectively.

Figure 5c represents the locus of the Joule’s heat capacity $R_{J} \left( I \right)$ against $x$ at 15 T, where the plot draws a quite similar locus to the theoretical one expected in Fig. 3c. Then, the calorimeter constants $C_{sys}$ and $C_{sys}^{*}$, and the molar excess heat $\gamma_{col} Q_{ann}$ were individually calculated.

**Figure 6:**
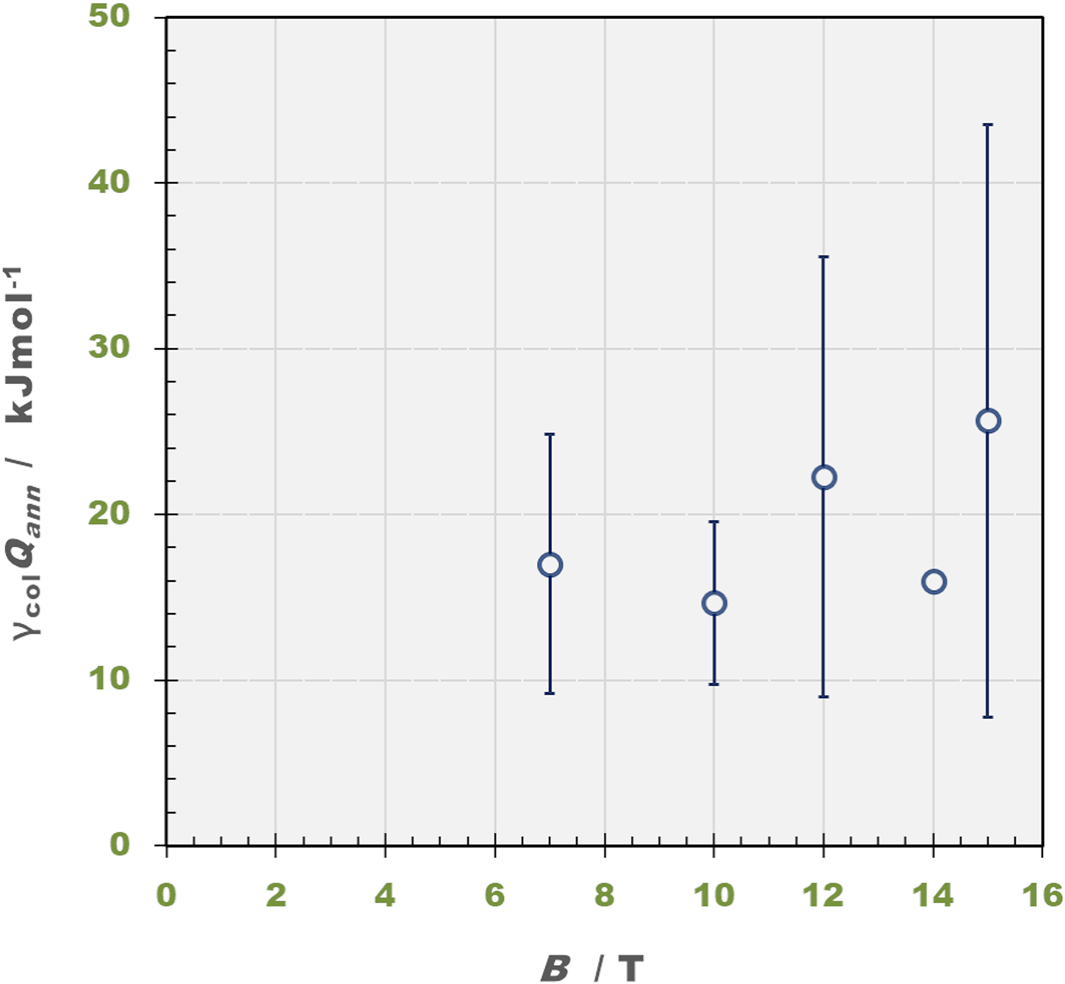
Plot of molar excess heat vs. magnetic flux density. $\gamma_{col} Q_{ann}$, molar excess heat; $B$, magnetic flux density. Experimental conditions are the same as those in Fig. 5.

In Fig. 6, the observed molar excess heats are plotted against magnetic flux densities. Beyond 7 T, it slightly increases with magnetic flux density, reaching ca. 25 kJ·mol^−1^ in average at 15 T, which is ca. 20% of the theoretical value of 112 kJ·mol^−1^. From Eq. (3), the collision efficiency $\gamma_{col} \left( { \pm 1} \right)$ is determined 0.22, where $\pm 1$ implies the single unit charge of the vacancy. In comparison with the case of copper redox reaction where $\gamma_{col} \left( { \pm 2} \right) \approx$ 1.0 was estimated, where $\pm 2$ implies the two unit charges of the vacancy, such a low collision efficiency may be attributed to a small radius of vacancy core of 0.32 nm, whereas the core radius of the vacancy created in copper redox reaction is 0.65 nm. Since the collision efficiency is assumed to be in proportion to the cross section of the vacancy core, we find a good relationship between the ratios of the collision efficiencies and the cross sections; $\gamma_{col} \left( { \pm 1} \right)/\gamma_{col} \left( { \pm 2} \right)\left( { = 0.22} \right) \approx$ 0.32^2^/0.65^2^ (= 0.24). On the other hand, below 7 T, we could not obtain the locus of the Joule’s heat capacity in the middle current range, but obtain only the locus in the low current range. This means that since due to weak Lorentz force, the mixing of the initial low- and high-temperature phases was insufficient.

## Discussion

Energy of matter activated by a 10 T magnetic field is so small only of the order of 1 milli eV that we can neglect it in comparison with the irreversible energy increments arising from MHD flow, which we can classify into kinetic energy of the flow, dissipated friction heat and the work of the pressure difference between the outlet and inlet of MHDE. However, all of them are estimated about one-thousandth times smaller than the Joule’s heat production^1^. On the other hand, reversible heats are generated at the electrode/electrolyte junction by electrochemical reaction. Other reversible heats except for the change in partial molar entropy of the half-cell reaction come from the interactions between heat and mass transport in the electrode (i.e., the thermocouple or electronic Seebeck effect) and the electrolyte (i.e., the thermal diffusion or Soret effect)^26,27^. However, as shown in the present case, if the same electrode and metal lead are used in each half cell, the electric transport-related terms are cancelled^28^. Owing to the isothermal condition maintained by MHD flow, we can also disregard the electrolytic transport-related terms. As a result, we can calculate the reversible heat for the whole cell reaction simply from the entropy change of reaction, i.e., $T\Delta_{R} S_{R}$^11^. However, in the redox reaction shown in Eqs. (1) and (2), the reversible heats of the half cell reactions are cancelled out each other, i.e., $T\Delta_{R} S_{R} = 0$. Namely, heat production except for Joule’s heat would not be observed, if vacancy annihilation were neglected.

In the ferricyanide–ferrocyanide redox reactions in potassium chloride solution without hydrogen and oxygen evolution by using the MHD flow in a c-type Pt MHDE, as shown in Fig. 5b, we observed at most a 2.3 °C (2.3 K) temperature increase of the system, which was translated into 6.0 °C (6.0 K) by the compensation of the escaping heat from it. Then, as shown in Fig. 5c, by applying linear regression to the locus of $R_{J} \left( I \right)$ in the middle current range, the molar excess heat $\gamma_{col} Q_{ann}$ and the calorimeter constant $C_{sys}$ were determined.

The accuracy and precision of Joule’s heat capacity method was firstly examined by the standard additive method. As a result, the accuracy was $\varepsilon_{a}$ = 78.3 ~ 86.0% and the precision was $\sigma_{p}$ = 61.3 ~ 65.4%, which means that instead of large deviation, the average is in good agreement with the standard value. This result ensures the validity of this method. Then, the excess heat from the collision of a pair of ionic vacancies was determined, which, beyond 7 T, gives rise to the values up to 25 kJ·mol^−1^ in average at 15 T, i.e., about 10% of the heat production by the combustion of hydrogen molecule 285.8 kJ·mol^−1^ at 25 °C, 1 bar. Then, the low collision efficiency $\gamma_{col} \approx 0.22$ is attributed to the small size of the vacancy core, i.e., $R^{*} = 0.32 \,nm$. However, below 7 T, we could not obtain the excess heat $\gamma_{col} Q_{ann}$. This means that the induced Lorentz force is too small to collide the vacancies.

In conclusion, the solvation energy stored in vacancy cores is liberated in the pair annihilation as excess heat by the neutralization of oppositely charged ionic clouds. In the present case of vacancy with a single unit charge, since the core radius is small, i.e., 0.32 nm, the theoretical molar excess heat is estimated 112 kJ·mol^−1^, and the collision efficiency is low, i.e., 0.22 at 15 T, so that the liberated energy was not so high, i.e., 25 kJ mol^−1^ in average at 15 T. However, in the case of vacancy with two-unit charges in copper redox reaction, the theoretical molar excess heat is 410 kJ·mol^−1^, and due to the twice larger radius, we obtained great excess heat production with much higher collision efficiency almost equal to 1.0. From these two examples of the excess heat production, we declare that the excess heat production by the pair annihilation of ionic vacancies is a universal phenomenon in electrode reaction. Therefore, the recycling of the wasted thermal energy of ionic vacancies in electrochemical industry would greatly contribute to a contemporary energy issue for global economy and ecology.

## Methods

The experimental method is the same as the previous report^1^ except for the electrode material and electrolyte solution. Ferricyanide-ferrocyanide redox reaction was performed in a 300 mol·m^−3^ K_3_[Fe(CN)_6_] + 300 mol·m^−3^ K_4_[Fe(CN)_6_] + 100 mol·m^−3^ KCl solution. Water was prepared by a pure water production system (Merck, Milli-Q). The reagents were in analytical grade (Wako pure chemical Co.). MHDE was composed of a channel of acrylic acid resin with two open ends; the channel was 10 mm high, 5 mm wide and 23 mm long. A pair of rectangular Pt electrodes (10 $\times$ 20 $\times$ 0.2 mm, Nilaco Co., 99.96% purity) working as cathode and anode were imbedded on the inner side walls at a distance of 5 mm. After confirming that the influence of magnetic field up to 15 T was below the environmental thermal disturbance, two thermal sensors (T type thermocouple) were attached to the outside of the channel and the bore space, of which leads were connected to a measuring instrument (KEYENCE Co., NR-600 with NR-TH08 unit). Then, as shown in Fig. 4a, the MHDE was set in a glass tube containing an electrolytic solution of 7.5 cm^3^ (c-type MHDE), and the whole electrode system was settled in the bore space of 15 T-cryocooled superconducting magnet at the High Field Laboratory for Superconducting Materials, Institute for Materials Research, Tohoku University. The solution flow in the MHDE was optically observed by a microscope (AnMo Electronics Co., Dino-Lite Premier2 S-DINOAD7013MT) from the bottom of the bore. For the standard additive method to assess the accuracy and precision of this measurement, the amount of the solution was tentatively changed. Then, the calorimeter constants of different amounts of the solution were measured. Sweeping the electrolysis current $I$ in a rate of 0.05 mA·s^−1^ from 0 A to 0.2 A with a potentiostat (Toho Technical Research Co., Ltd., PS-2000) in galvanostatic mode, we measured the potential response $\Delta V$ between the cathode and anode of the c-type MHDE. The electrode potentials of cathode and anode were measured by the tentative reference electrode of a platinum rod of 0.2 mm diameter. During the experiment, the temperatures of the electrodes, the solution and the bore space were measured. After attaining the upper limit, to measure the heat escaping from the electrode system, the current was switched off, and decreasing temperature of the solution was recorded by a personal computer.

## Supplementary information

Supplementary Information.

## References

[1] Miura M (2019). Excess heat production by the pair annihilation of ionic vacancies in copper redox reactions. Sci. Rep..

[2] Aogaki R (2016). Origin of nanobubbles electrochemically formed in a magnetic field: ionic vacancy production in electrode reaction. Sci. Rep..

[3] Aogaki R (2008). Theory of stable formation of ionic vacancy in a liquid solution. Electrochemistry.

[4] Sugiyama A (2016). Lifetime of ionic vacancy created in redox electrode reaction measured by cyclotron MHD electrode. Sci. Rep..

[5] Sugiyama A (2013). Non-electrochemical nanobubble formation in ferricyanide/ferrocyanide redox reaction by the cyclotron effect under a high magnetic field. Electrochemistry.

[6] Miura M (2014). Microbubble formation from ionic vacancies in copper electrodeposition under a high magnetic field. Electrochemistry.

[7] Oshikiri Y (2015). Microbubble formation from ionic vacancies in copper anodic dissolution under a high magnetic field. Electrochemistry.

[8] Miura M (2017). Magneto-dendrite effect: Copper electrodeposition under high magnetic field. Sci. Rep..

[9] Aogaki R (2009). Origin of nanobubble-formation of stable vacancy in electrolyte solution. ECS Trans..

[10] Newman, J. & Thomas-Alyea, K. E. *Electrochemical Systems, 3rd edition Ch. 13* (Wiley, Hoboken, 2004).

[11] Thomas KE, Newman J (2003). Thermal modeling of porous insertion electrodes. J. Electrochem. Soc..

[12] Fahidy TZ (1976). Wave phenomena in magnetoelectrolytic systems. Electrochim. Acta.

[13] Mohanta S, Fahidy TZ (1976). The hydrodynamics of a magnetoelectrolytic cell. J. Appl. Electrochem..

[14] Aaboubi O (1990). Magnetic field effects on mass transport. J. Electrochem. Soc..

[15] Olivier A, Merienne E, Chopart JP, Aaboubi O (1992). Thermoelectrochemical impedances: A new experimental device to measure thermoelectrical transfer functions. Electrochim. Acta.

[16] Fahidy, T. Z. *The Effect of Magnetic Fields on Electrochemical Processes*, *Modern Aspects of Electrochemistry, No. 32 Ch. 5* (Kluwer Academic/Plenum Publishers, New York, 1999).

[17] Alemany A, Chopart JP (2007). Magnetohydrodynamics, Historical Evolution and Trends.

[18] Monzon LMA, Coey JMD (2014). Magnetic fields in electrochemistry: The Lorentz force. A mini-review. Electrochem. Commun..

[19] Aogaki, R. & Morimoto, R. *Heat and Mass Transfer—Modelling and Simulation Ch. 9* (Intech, Rijeka 2011). https://www.intechopen.com/books/heat-and-mass-transfer-modeling-and-simulation/nonequilibrium-fluctuations-in-micro-mhd-effects-on-electrodeposition.

[20] Morimoto R (2019). Theory of microscopic electrodeposition under a uniform parallel magnetic field—1. Nonequilibrium fluctuations of magnetohydrodynamic (MHD) flow. J. Electroanal. Chem..

[21] Morimoto R (2019). Theory of microscopic electrodeposition under a uniform parallel magnetic field—2. Suppression of 3D nucleation by micro-MHD flow. J. Electroanal. Chem..

[22] Aogaki R, Fueki K, Mukaibo T (1975). Application of magnetohydrodynamic effect to the analysis of electrochemical reactions. 2. Diffusion process in MHD forced flow of electrolyte solution. Denki kagaku (presently Electrochemistry).

[23] Aogaki R, Fueki K, Mukaibo T (1976). Diffusion process in viscous-flow of electrolyte solution in magnetohydrodynamic pump electrodes. Denki kagaku (presently Electrochemistry).

[24] Boum GBN, Alemany A (1999). Numerical simulations of electrochemical mass transfer in electromagnetically forced channel flows. Electrochim. Acta.

[25] Kambe T, Drazin PG (1998). Fluid Dynamics-Stability and Turbulence.

[26] Newman J (1995). Thermoelectric effects in electrochemical systems. Ind. Eng. Chem. Res..

[27] Lewis GN, Randall M, Pitzer KS, Brewer L (1961). Thermodynamics.

[28] Agar, J. N. *Advances in Electrochemistry and Electrochemical Engineering, Vol. 3, Thermogalvanic Cells* (Wiley, New York, 1963).

