# Supplementary material for: Excess heat production in the redox couple reaction of ferricyanide and ferrocyanide
Source: Sci Rep. 2020 Nov 18;10:20072. doi: 10.1038/s41598-020-76611-3 (PMC7674507; doi:10.1038/s41598-020-76611-3)
Supplement: Supplementary file 1 — Supplementary Information. [file 41598_2020_76611_MOESM1_ESM.docx]

**Appendixes for**

**Excess heat production in the redox couple reaction of ferricyanide and ferrocyanide**

Atsushi Sugiyama ^1, 2, 3*^, Makoto Miura ^4^, Yoshinobu Oshikiri ^5^, Yena Kim ^3^,

Ryoichi Morimoto ^6^, Miki Miura ^7^, Tetsuya Osaka ^2^, Iwao Mogi ^8^, Yusuke Yamauchi ^3, 9*^,

and Ryoichi Aogaki ^3, 10*^

*^1^Yoshino Denka Kogyo, Inc., Japan, ^2^Waseda Univveristy Research Organization for Nano & Life Innovation, Japan, ^3^National Institute for Materials Science, Japan, ^4^Hokkaido Polytechnic College, Japan, ^5^Yamagata College of Industry and Technology, Japan, ^6^Saitama Industrial Technology Center, Japan, ^7^Polytechnic Center Kimitsu, Japan, ^8^IMR, Tohoku University, Japan, ^9^Universiry of Queensland, Commonwealth of Australia, ^10^Polytechnic University, Japan*

**Appendix A**

*Modification of measured temperature difference.* From the foregoing paper [1], the heat balance equation in a quasi-adiabatic redox reaction system is expressed by

| $C_{sys}\frac{d\Delta T^{*}}{dt}=\Delta VI+\frac{\gamma_{col}Q_{ann}}{nF}I$ | (A.1) |
| --- | --- |

where $C_{sys}$ is the calorimeter constant (unit: J·K^-1^), i.e., the effective heat capacity of the system, $t$ is the time (unit: s), $\Delta T^{*}$ is the temperature difference (unit: K) between the system $T\left( t \right)$ and the environment $T_{a}\left( t \right)$, where the escaping heat from the system is compensated as mentioned below, $\Delta V (>0)$ is the cell voltage (unit: V), $\gamma_{col}$ is the collision efficiency, and $Q_{ann}$ is the molar excess heat (unit: J·mol^-1^). Then, $n$ is the electron number transferring in the cell reaction, $F$ is Faraday constant (96485 C·mol^-1^), and $I$ is the electrolytic current (unit: A). In view of redox cell reaction such as ferricyanide-ferrocyanide redox reaction, the molar reaction entropy $\Delta_{R}S_{R}=0$ is assumed.

To calculate the excess heat by the pair annihilation, the positive current $I$ increasing with time $t$ is used.

| $for$ | (A.2) |
| --- | --- |

where $a$ is the positive sweep rate (unit: A·s^-1^). Substituting Eq. (A.2) into Eq. (A.1), we integrate the resultant equation from $t=0$ to $t=t$, and obtain the following basic heat balance equation.

| $C_{sys}\left( \Delta T^{*}-\Delta T_{0}^{*} \right)\left( =Q_{tot} \right)=\frac{1}{a}\int_{0}^{I} \Delta VIdI+\frac{\gamma_{col}Q_{ann}}{2nFa}I^{2} for$ | (A.3) |
| --- | --- |

where $\Delta T_{0}^{*}$ is the initial value of $\Delta T^{*}$, and $Q_{tot}$ is the total heat stored in the system (unit: J).

For compensating the escaping heat from the cell system, under isothermal condition, directly measured temperature difference $\Delta T$ is modified by the following equation [1]

| $\Delta T^{*}=\Delta T+\alpha\int_{0}^{t} \Delta Tdt$ | (A.4) |
| --- | --- |

where $\alpha$ is the time constant of the escaping heat from the system (unit: s^-1^).

As shown in Fig. A1, a glass vessel of MHDE filled with solution and N_2_ gas is placed in the magnet bore. In the early stage of current sweeping, due to weak Lorentz force, the effect of the MHD flow is approximately disregarded, i.e., initially the solution is assumed in keeping stationary, and a thermal sensor attached on the MHDE would indicate a slightly rising temperature with time.

However, if the bore temperature starts to rise, on account of large difference of the heat conductivities between N_2_ gas (low) and glass vessel (high), an adverse temperature gradient occurs in the solution by warming the underside. Due to thermal expansion, the solution at the bottom will be lighter than the solution at the top; and this is a top-heavy arrangement which is potentially unstable. Because of this instability assisted by the Lorentz force, the less warm part of the solution at the top will be overturned with the warmer part at the bottom. In accordance with the redistribution of temperature, the measured temperature $T\left( t \right)$ by the thermal sensor is once lowered then raised, taking a minimum temperature $T\left( t_{m} \right)$ at a time $t=t_{m}$. In case of a small amount of excess heat production like the present case, such a convection makes serious systematic error in the temperature measurement. To prevent it, we should control the bore temperature to be a little lower than the measured temperature, and to slowly decrease with time.

However, actually, it is difficult to precisely control the bore temperature. To improve such a problem, the following procedure is taken: The minimum value $\Delta T_{0m}$ is firstly defined by the difference between the initial temperature $T\left( 0 \right)$ and the minimum temperature $T\left( t_{m} \right)$.

| $\Delta T_{0m}=T\left( 0 \right)-T\left( t_{m} \right) \left( >0 \right)$ | (A.5) |
| --- | --- |

As will be discussed in Appendix B, at the initial stage of the current sweeping, Joule’s heat is dominant, so that the measured temperature must monotonously increase with the current in the absence of natural convection. In accordance with such a behavior of the temperature, we secondarily modify the measured temperature $T\left( t \right)$ as follows,

| $\tilde{T}\left( t \right)=T\left( 0 \right) for 0\leq t\leq t_{m}$ | (A.6) |
| --- | --- |
| $\tilde{T}\left( t \right)=T\left( t \right)+\Delta T_{0m} for t>t_{m}$ | (A.7) |

where the modified temperature $\tilde{T}\left( t \right)$ is smoothly connected at the minimum point $t=t_{m}$. In the absence of the minimum point without natural convection, there is no modification, i.e.,

| $\tilde{T}\left( t \right)=T\left( t \right) for t\geq0$ | (A.8) |
| --- | --- |

The modified temperature difference $\Delta\tilde{T}\left( t \right)$ is obtained by subtracting the ambient bore temperature $T_{a}\left( t \right)$ from the modified temperature $\tilde{T}\left( t \right)$.

| $\Delta\tilde{T}\left( t \right)\equiv\tilde{T}\left( t \right)-T_{a}\left( t \right)$ | (A.9) |
| --- | --- |

where $T_{a}\left( t \right)$ is a weak function of time.

Finally, by replacing $\Delta T^{*}-\Delta T_{0}^{*}$ with $\Delta T^{*}$, Eq. (A.3) is expressed by

| $C_{sys}\Delta T^{*}\left( =Q_{tot} \right)=\frac{1}{a}\int_{0}^{I} \Delta VIdI+\frac{\gamma_{col}Q_{ann}}{2nFa}I^{2} for I\geq0$ | (A.10) |
| --- | --- |

where in terms of the modified temperature difference $\Delta\tilde{T}\left( t \right)$, $\Delta T^{*}$ in Eq. (A.4) is simply rewritten as

| $\Delta T^{*}=\Delta\tilde{T}\left( t \right)-\Delta\tilde{T}\left( 0 \right)+\alpha\int_{0}^{t} \Delta\tilde{T}\left( t \right)dt$ | (A.11) |
| --- | --- |

The revised $\Delta T^{*}$ in Eq. (A.11) therefore starts from $0$.


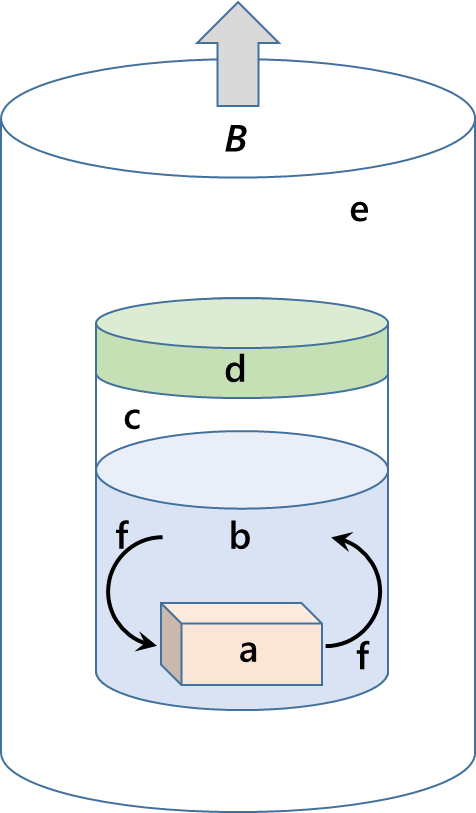


**Figure A1 | Overturn of the solution induced by thermal instability and weak Lorentz force.**

a, MHDE; b, solution phase; c, gas phase; d, cover of the vessel; e, bore space of magnet, *B*, Magnetic flux density; f, arrows indicate the convection.

**Appendix B**

*Joule’s heat capacity method for calorimetry experiment.* As shown in Eq. (A.10), for a redox reaction, the heat balance equation of the cell system is expressed by

| $Q_{tot}=Q_{Joule}+Q_{excess}$ | (B.1) |
| --- | --- |

where $Q_{tot}$ is the total heat stored in the system (unit: J), represented by

| $Q_{tot}\equiv C_{sys}\Delta T^{*}$ | (B.2) |
| --- | --- |

where $C_{sys}$ (unit: J·K^-1^) is the calorimeter constant of the system, and $\Delta T^{*}$is the compensated total temperature difference (unit: K) defined by Eq. (A.11). $Q_{Joule}$ is Joule’s heat (unit: J) defined by

| $Q_{Joule}\equiv\frac{1}{a}\int_{0}^{I} \Delta VIdI$ | (B.3) |
| --- | --- |

where $\Delta V$ is the cell voltage (unit: V), $a$ is the sweep rate of the current (unit: A·s^-1^), and $Q_{excess}$ is the produced excess heat (unit: J) represented by

| $Q_{excess}\equiv\frac{\gamma_{col}Q_{ann}}{2nFa}I^{2}$ | (B.4) |
| --- | --- |

where $Q_{ann}$ is the molar excess heat of the pair annihilation (unit: J·mol^-1^), and $\gamma_{col}$is the collision efficiency, $n$ is the positive charge number transferring in the cell reaction, and $F$ is Faraday constant.

*a. Low current range (Two-temperature-phase state)*. In the early stage of the current sweep, small electrolytic current induces a weak laminar MHD flow, only leading to the small collision efficiency of the vacancies, i.e., $\gamma_{col}\approx0$, and the resultant small excess heat production, $Q_{excess}\approx0$. Namely, in the low current range, we cannot measure the excess heat production. As a result, the stored total heat $Q_{tot}$ is approximately equal to the produced Joule’s heat,

| $Q_{tot}\approx Q_{Joule}$ | (B.5) |
| --- | --- |

Here, due to the weak laminar MHD flow, separation of the two phases is maintained, so that the total heat stored by the system $Q_{tot}$ is exactly expressed by the heats stored in the low- and high-temperature phases (unit: J), $Q_{l}$ and $Q_{h}$, respectively.

| $Q_{tot}=Q_{l}+Q_{h}$ | (B.6) |
| --- | --- |

where

| $Q_{l}\equiv C_{sys,l}\Delta T_{l}^{*}$ | (B.7) |
| --- | --- |

and

| $Q_{h} \equiv C_{sys, h}\Delta T_{h}^{*}$ | (B.8) |
| --- | --- |

where $C_{sys,l}$ and $C_{sys,h}$ are the calorimeter constants of the low- and high-temperature phases (unit: J·K^-1^), respectively, which are effective heat capacities of the two phases involving solution, sensor, vessel and electrode.$\Delta T_{l}^{*}$ and ${\Delta T}_{h}^{*}$ are the temperature differences at the low- and high-temperature phases (unit: K), respectively. It should be noted that as shown in Fig. 3b (1), $\Delta T_{l}^{*}$ is only a measurable quantity between them. By means of the measured $\Delta T_{l}^{*}$, we can determine the stored heat $Q_{l}$ of the low-temperature phase.

However, due to the non-measurable temperature difference$\Delta T_{h}^{*}$of the high-temperature phase, $Q_{h}$ is also non-measurable. Substituting Eq. (B.5) into Eq. (B.6), we have

| $Q_{Joule}\approx Q_{l}+Q_{h}$ | (B.9) |
| --- | --- |

Here, we introduce the effective Joule’s heat capacity $R_{J}\left( I \right)$ (unit: J·K^-1^) as a measurable quantity.

| $R_{J}\left( I \right)\equiv Q_{Joule}/\Delta T_{l}^{*} for \Delta T_{l}^{*}>0$ | (B.10) |
| --- | --- |

The value of $R_{J}\left( I \right)$ can be calculated only by the measurable quantities, the Joule’s heat $Q_{Joule}$ and the measured temperature difference $\Delta T_{l}^{*}$. In accordance with Eq. (A.11), $\Delta T_{l}^{*}$ starts from $0$, so that to prevent the value of $R_{J}\left( I \right)$ from large fluctuation, the span of the sampling points of $\Delta T_{l}^{*}$ should be taken at a sufficient time intervals as much as possible.

Considering that the excess heat in Eq. (B.4) is in proportion to the 2nd power of the

current $I^{2}$, as another measurable quantity, we introduce the variable $x$ (unit: A^2^K^-1^), as follows.

| $x \equiv I^{2}/\Delta T_{l}^{*}$ | (B.11) |
| --- | --- |

Substituting Eqs. (B.7), (B.10) and (B.11) into Eq. (B.9), we obtain

| $R_{J}\left( I \right)\approx C_{sys,l}+Q_{h}^{*}x$ | (B.12) |
| --- | --- |

where the slope $Q_{h}^{*}$ (unit: J·A^-2^) is defined by

| $Q_{h}^{*} \equiv Q_{h}/I^{2} (>0)$ | (B.13) |
| --- | --- |

At the early stage of the current sweep, as mentioned above, the whole system is separated by the low- and high-temperature phases. Since the heat source is involved in the high-temperature phase, according to Eq. (B.5), the high-temperature phase is mainly heated by the Joule’s heat $Q_{Joule}$, whereas due to the weak MHD flow, the low-temperature phase hardly receives such heat.

| $Q_{h}\approx Q_{Joule} and Q_{l}\approx0$ | (B.14) |
| --- | --- |

The equation $Q_{l}\approx0$ indicates that the measured temperature difference $\Delta T_{l}^{*}$is approximately kept constant, so that the variable *x* in Eq. (B.11) increases with sweeping current $I$. The relationship $Q_{h}\approx Q_{Joule}$ is substituted into Eq. (B.13). As will be discussed later, in view of the fact that Joule’s heat is approximately expressed by $Q_{Joule}\propto I^{3}$ in Eq. (B.23), we can estimate the slope $Q_{h}^{*}$ as

| $Q_{h}^{*}\approx Q_{Joule}/I^{2}\propto I (>0)$ | (B.15) |
| --- | --- |

Namely, the positive slope $Q_{h}^{*}$ also increases with $I$. This implies that in the low current range, the locus of the Joule’s heat capacity $R_{J}\left( I \right)$ against $x$ draws a rising curve with an increasing slope.

*b. Middle current range (Quasi-isothermal state)*. With current increasing, Lorentz force is strengthened, so that the laminar solution flow is changed into transient flow with Kármán’s vortexes shown in Fig. 3b (2). At the same time, the collision efficiency of the vacancy $\gamma_{col}$ increases, and the excess heat production $Q_{excess}$ by the pair annihilation is greatly promoted. The mixing of the low- and high-temperature phases occurs simultaneously, resulting in the decrease of the thickness $\delta$ of the low-temperature phase down to the order of the size of the thermal sensor $\mathcal{l}$. The mixture however does not attain the perfect molecular mixing, but is kept in a quasi-molecular mixing state where numerous low- and high-temperature sub-phases with the small volumes of $\Delta v_{l,i}$ and $\Delta v_{h,j}$ (for $i, j=1, 2, 3, ....$) of the order of length of $\mathcal{l}$ are mingled with each other, so that the heat transfer between the sub-phases is much faster than the rise of measured temperature, forming a quasi-isothermal state. The total volumes of the two phases are $v_{l} \left( =\sum\Delta v_{l,i} \right)$ (unit: cm^3^) and $v_{h} \left( =\sum\Delta v_{h,j} \right)$ (unit: cm^3^), respectively. The whole volume of the solution $v$ is therefore expressed by

| $v=v_{l}+v_{h}$ | (B.16) |
| --- | --- |

The total heat stored in the whole volume of the solution is expressed by $c_{sol}\rho\left( v_{l}\Delta T_{l}^{*}+v_{h}\Delta T_{h}^{*} \right)$, where $c_{sol}$ is the specific heat of the experimental solution containing electrolytes (unit: J·g^-1^·K^-1^), and $\rho$ is the density of the solution (unit: g·cm^-3^), where since the temperature change is small, the density is assumed constant.

Here, due to quasi-isothermal state, the heat amount of the solution measured by the thermal sensor $c_{eff,mc}\rho v\Delta T_{l}^{*}$ is equalized to the stored total heat.

| $c_{eff,mc}\rho v\Delta T_{l}^{*}=c_{sol}\rho\left( v_{l}\Delta T_{l}^{*}+v_{h}\Delta T_{h}^{*} \right)$ | (B.17) |
| --- | --- |

where $c_{eff,mc}$is the effective specific heat of the experimental solution (unit: J·g^-1^·K^-1^) measured by the temperature difference $\Delta T_{l}^{*}$ in the middle current range. To express the quasi-isothermal state, the average temperature difference ${\Delta T}_{av}^{*}$ of the mixed phase (unit: K) is newly introduced as follows,

| $\Delta T_{av}^{*} \equiv\left( v_{l}\Delta T_{l}^{*}+v_{h}\Delta T_{h}^{*} \right)/v$ | (B.18) |
| --- | --- |

Substituting Eq. (B.18) into Eq. (B.17), we get

| $c_{eff,mc}/c_{sol}=\Delta T_{av}^{*}/\Delta T_{l}^{*}$ | (B.19) |
| --- | --- |

Namely, the ratio of $c_{eff,mc}$to $c_{sol}$ is equal to the ratio of $\Delta T_{av}^{*}$ to $\Delta T_{l}^{*}$, so that the more the excess heat is generated, the larger the $c_{eff,mc}$ is. Since the total heat stored in the system is measured by the temperature in the low temperature phase, the effective heat capacity $c_{eff,mc}$ is always larger than the actual one $c_{sol}$.

Under the quasi-isothermal condition, with the measurable temperature difference $\Delta T_{l}^{*}$, Eq. (B.1) is rewritten as

| $C_{sys}\Delta T_{l}^{*}=Q_{Joule}+Q_{excess}$ | (B.20) |
| --- | --- |

In the same way as Eq. (B.12), the Joule’s heat capacity $R_{J}\left( I \right)$ is thus represented by

| $R_{J}\left( I \right)=C_{sys}-\frac{\gamma_{col}Q_{ann}}{2nFa}x$ | (B.21) |
| --- | --- |

As the MHD flow is promoted, the collisions of ionic vacancies with opposite charges drastically increase. The excess heat production is activated together with the mixing between the two phases, so that the temperature difference $\Delta T_{l}^{*}$ of the low-temperature phase involving the thermal sensor greatly increases, and the variable $x$ ($\equiv I^{2}/\Delta T_{l}^{*}$) resultantly decreases. Due to the linear function of *x* with a negative slope, $R_{J}\left( I \right)$ increases with decreasing $x$.

*c. High current range (Isothermal state)* In the high current range, as the Lorentz force increases, as shown in Fig. 3b (3), the transient MHD flow is furthermore changed into turbulent flow with micro-vortexes. As a result, a perfect mixing state, i.e., isothermal state emerges, where the measured temperature difference $\Delta T_{l}^{*}$ and also $\Delta T_{h}^{*}$ become equal to that of the solution $\Delta T^{*}$. Here, the Joule’s heat is, as shown in Eq. (B.3), calculated by the integral of the product of the cell voltage and the current, $\Delta VI$. The cell voltage in the high current range is approximately expressed by Ohm’s law. Assuming $R_{s}$ as a solution resistance, and neglecting the constant term, we can represent the cell voltage as follows.

| $\Delta V\approx R_{s}I$ | (B.22) |
| --- | --- |

Substituting Eqs. (A.2) and (B.22) into Eq. (B.3), we obtain

| $Q_{Joule}\approx\left( R_{s}/3a \right)I^{3}$ | (B.23) |
| --- | --- |

Namely, Joule’s heat $Q_{Joule}$ is approximately in proportion to the 3rd power of $I$. On the other hand, excess heat $Q_{excess}$ is, as shown in Eq. (B.4), in proportion to the 2nd power of $I$. This means that in the high current range, Joule’s heat always prevails over excess heat. In view of the fact that the perfect mixing is attained, and the low- and high-temperature phases are unified to a single phase of a temperature difference $\Delta T^{*}$, $\Delta T_{av}^{*}=\Delta T_{l}^{*}\left( =\Delta T^{*} \right)$ from Eq. (B.19), the effective specific heat of the solution measured in the high current range $c_{eff,hc}$ (unit: J·K^-1^·g^-1^) is equal to the specific heat of the experimental solution $c_{sol}$.

| $c_{eff,hc}=c_{sol}$ | (B.24) |
| --- | --- |

From Eqs. (B.19) and (B.24), we have the following relationship between $c_{eff,mc}$and $c_{eff,hc}$.

| $c_{eff,hc}<c_{eff, mc}$ | (B.25) |
| --- | --- |

Therefore, the effective specific heat of the solution obtained in the middle current range is always larger than that in the high current region. From Eq. (B.25), we can derive a conclusion that the calorimeter constant $C_{sys}$ in the middle current range is larger than $C_{sys}^{*}$ in the high current region.

| $C_{sys}^{*}<C_{sys}$ | (B.26) |
| --- | --- |

Due to dominant Joule’s heat, Eq. (B.20) is rewritten as

| $C_{sys}^{*}\Delta T^{*}=Q_{Joule}$ | (B.27) |
| --- | --- |

where $C_{sys}^{*}$ is the calorimeter constant in the high current range. Equation (B.27) indicates that in the high current range, the locus of $R_{J}\left( I \right)$ takes a constant value of $C_{sys}^{*}$.

| $R_{J}\left( I \right)=C_{sys}^{*}$ | (B.28) |
| --- | --- |

That is, in the high current range, we cannot measure the excess heat production again.

**Appendix C.**

*The accuracy and precision of Joule's heat capacity method.* As discussed in Appendix

B, the effective specific heat $c_{eff,hc}$measured in the high current range is consistent with the specific heat of the experimental solution $c_{sol}$.

| $c_{eff,hc}=c_{sol}$ | (B.24) |
| --- | --- |

As a result, in terms of the standard additive method adopting the experimental solution as a reference material, we can assess the accuracy and precision of the present method in the following: Drawing the locus of $R_{J}\left( I \right)$, we first determine the calorimeter constant $C_{sys}^{*}$ in the high current range. Then, drawing the locus again after adding the extra solution of a mass $\Delta m_{sol}$ (unit: g), we obtain the increments of the calorimeter constant $\Delta C_{sys}^{*}$, which is equal to the effective heat capacity $c_{eff,hc}\Delta m_{sol}$. Comparing the plot of $\Delta C_{sys}^{*}$ vs. $\Delta m_{sol}$ with the plot of the standard value, we can ascertain the relationship in Eq. (B.24) (see Fig. 4b). By dividing the ${\Delta C}_{sys}^{*}$ with $\Delta m_{sol}$, each $c_{eff,hc}$ is obtained. With the obtained data, the average value $\left\langle c_{eff,hc} \right\rangle$ and the standard deviation $\Delta c_{eff,hc}$ are calculated. Assuming that sufficiently suitable value is regarded as the standard value, we can calculate the accuracy of this method defined as the degree of the agreement of the average value with the standard value.

| $\varepsilon_{a}\equiv\left( 1-\left\vert c_{stand}-\left\langle c_{eff,hc} \right\rangle\right\vert/c_{sol} \right)\times100\%$ | (C.1) |
| --- | --- |

where $c_{stand}$ is the standard value of the specific heat. The precision of this method is defined as the degree of the deviation from the average value.

| $\sigma_{p}\equiv\Delta c_{eff,hc}/c_{stand}\times100\%$ | (C.2) |
| --- | --- |

In the middle current range of the locus of $R_{J}\left( I \right)$, we can measure another calorimeter constant $C_{sys}$, which gives rise to the effective specific heat of the solution in the middle current range $c_{eff,mc}$. By using the standard value mentioned above, Eq. (B.19), in Appendix B is rewritten as

| $c_{eff,mc}/c_{stand}=\Delta T_{av}^{*}/\Delta T_{l}^{*}$ | (C.3) |
| --- | --- |

where $\Delta T_{l}^{*}$ is the temperature difference in the low temperature phase of the solution, and $\Delta T_{av}^{*}$ is the average temperature difference of the whole solution. Therefore, from the comparison of the measured $\Delta C_{sys}$value with the standard value, we can estimate the ratio of $\Delta T_{av}^{*}$ to $\Delta T_{l}^{*}$ (see Fig. 4c). The result shown in Fig. 4c is the experimental evidence of existence for the quasi-isothermal state in Fig. 3b (2).
